# Movement response of small mammals to burn severity reveals importance of microhabitat features

**DOI:** 10.1093/jmammal/gyad117

**Published:** 2023-12-24

**Authors:** Sandy A Slovikosky, Melissa J Merrick, Marina Morandini, John L Koprowski

**Affiliations:** School of Natural Resources and the Environment, University of Arizona, 1064 E. Lowell Street, Tucson, AZ 85721, United States; Department of Biology, University of Oxford, 11a Mansfield Road, Oxford OX1 3SZ, United Kingdom; School of Natural Resources and the Environment, University of Arizona, 1064 E. Lowell Street, Tucson, AZ 85721, United States; Beckman Center for Conservation Research, San Diego Zoo Wildlife Alliance, 15600 San Pasqual Valley Road, Escondido, CA 92027, United States; School of Natural Resources and the Environment, University of Arizona, 1064 E. Lowell Street, Tucson, AZ 85721, United States; Natural Science Department, Paul Smith’s College, 7777 NY-30, Paul Smiths, NY 12970, United States; School of Natural Resources and the Environment, University of Arizona, 1064 E. Lowell Street, Tucson, AZ 85721, United States; Haub School of Environment and Natural Resources, University of Wyoming, 804 E. Fremont Street, Laramie, WY 82072, United States

**Keywords:** animal movement, burn severity, disturbance ecology, fluorescent powder tracking, *Neotoma mexicana*, path tortuosity, postfire response, small mammal, gravedad de las quemaduras, movimiento de animales, *Neotoma mexicana*, perturbación de la ecología, respuesta posterior al incendio de los pequeños mamíferos, seguimiento del polvo fluorescente, tortuosidad del camino

## Abstract

Disturbance events are increasing at a global scale, with cascading impacts to ecosystems and residents therein that include fragmentation and altered vegetation structure and composition. Such changes may disproportionately impact small mammal movements, risk perception, and community dynamics as smaller species perceive such changes at finer spatial scales. We examined movement response to burn severity, vegetation structure, and composition in Mexican woodrats (*Neotoma mexicana*), a common but understudied small mammal species. The study was conducted on Mt. Graham in southeastern Arizona, United States, following a fire that burned over 19,400 ha. We measured path tortuosity of woodrats translocated over patches of different burn severity. Tortuosity can indicate microhabitat selection, foraging behavior, and perceived predation risk—features affecting population-level processes that changes in community composition alone cannot fully demonstrate. We captured woodrats, released them 50 m away from their midden, and used fluorescent powder to track woodrat movement paths through areas of low–severe burn severity. We analyzed features of the resulting powder trails including straightness, average step length, fractal dimension, and squared displacement. We also compared used versus expected vegetation structure and composition along movement paths across burn severities. Analyses indicated shorter step length with increased bare ground, as well as higher squared displacement in areas with more logs. Vegetation analyses likewise showed that logs were heavily used in low-burned areas, whereas dense vegetation was avoided in highly burned areas. Burn severity alone did not have a direct effect on movement parameters, rather its influence on vegetative composition and structure appears to be most important. Selection for logs and avoidance of dense vegetation may be attributed to auditory concealment and ease of travel. With projected increases in wildfire extent and severity, this work represents an understudied approach to understanding these disturbances and their effects on ecological communities.

## Teaser

Small mammal behavior helps determine how disturbances, like fire, affect ecosystems. Woodrats use logs to move through burned areas, and more bare ground results in shorter segments of movement trails. Managing vegetation postdisturbance could thus be important for wildlife.

## Resumen

Los eventos de perturbación están aumentando a escala global, con alto impacto para los ecosistemas y sus residentes que incluyen fragmentación y alteración de la estructura y composición de la vegetación. Dichos cambios pueden afectar desproporcionadamente los movimientos de los pequeños mamíferos, la percepción de riesgo y la dinámica comunitaria, ya que las especies más pequeñas perciben dichos cambios en escalas espaciales más finas. Examinamos la respuesta del movimiento de acuerdo a la gravedad de las quemaduras, la estructura de la vegetación y la composición en los woodrat mexicanos (*Neotoma mexicana*), una especie del pequeño mamífero común poco estudiada. El estudio se llevó a cabo en el monte Graham, en el sureste de Arizona, USA, tras un incendio que quemó más de 19.400 ha. Medimos la tortuosidad del camino de los woodrat traslocados sobre parches de diferente gravedad de quemaduras. La tortuosidad puede indicar selección de microhábitat, comportamiento de búsqueda de alimento y riesgo percibido de depredación, características que afectan los procesos a nivel poblacional y que los cambios en la composición de la comunidad por sí solos no pueden demostrar completamente. Capturamos woodrat mexicanos, los liberamos a 50 m de su muladar y utilizamos polvo fluorescente para rastrear las rutas de movimiento que dejarían a través del área con quemaduras de gravedad baja y severa. Analizamos las características de los rastros de polvo fluorescente que dejaron los woodrat, incluido la rectitud, la longitud promedio del paso, la dimensión fractal y el desplazamiento al cuadrado. También comparamos la estructura y composición de la vegetación utilizada versus la esperada a lo largo de las rutas de movimiento a través de la gravedad de los incendios. Los análisis indicaron una longitud de paso más corto con mayor suelo desnudo, así como un mayor desplazamiento cuadrado en áreas con más troncos. Los análisis de vegetación también mostraron que se utilizaron muchos troncos en áreas poco quemadas, mientras que se eludía la vegetación densa en áreas muy quemadas. La gravedad de las quemaduras por sí sola no tuvo un efecto directo sobre los parámetros de movimiento, sino que su influencia sobre la composición y estructura vegetativa parece ser la más importante. La elección de troncos y el eludir vegetación densa pueden atribuirse a que los woodrat evitan hacer ruido para protegerse de los depredadores, y por la facilidad de viajar. Con los incendios forestales proyectados a aumentar en extensión y gravedad, este trabajo representa un enfoque poco estudiado para comprender las perturbaciones y sus efectos en las comunidades ecológicas.

Disturbance events are increasing at a global scale—with compounding impacts to ecosystems that include fragmentation, altered vegetation cover, structure, and composition, and changes in soil conditions and plant and animal species diversity—depending on the intensity and time since the disturbance (Avenant 2000; Pearce and Venier 2005; Leis et al. 2008). In the southwestern United States, forests are particularly sensitive to drought and increasing temperatures and many coniferous forests are experiencing a disturbance cascade driven by drought, temperature extremes, insect outbreaks, tree death, and fire (Williams et al. 2010; Millar and Stephenson 2015). Of particular concern is growing evidence that postdisturbance forest community types are likely to change or transition to novel vegetation communities in the face of increased climate stressors (Williams et al. 2013; Parks et al. 2019; Coop et al. 2020; O’Connor et al. 2020). For wildlife, disturbance-driven changes such as habitat loss or alteration, and novel vegetation community conversions may disproportionately impact habitat specialist species or guilds. Extreme environmental conditions, habitat fragmentation, and human activity can elicit changes in small mammal species richness, abundance, and behaviors (Avenant 2000; Vernes and Haydon 2001; Leis et al. 2008). Therefore, small mammals are useful indicators of such disturbance effects on wildlife because they perceive and respond to disturbance at fine spatial scales (Gehring and Swihart 2003), providing an early warning system that ecosystem function and community dynamics may be at risk. The effects of fire and fire severity on small mammals vary with the ecology of individual species. Burned areas generally contain lower rodent abundance than unburned areas; however, changes in species richness are more variable (Kirkland et al. 1996; Monroe et al. 2004; Horn et al. 2012; Griffiths and Brook 2014). Differences in habitat selection pre- and postfire from altered vegetation structure may occur as well, though influenced by season (Kaufman et al. 1988). Small mammal population and community responses to fire have received much attention; however, understanding the impact of fire and postfire habitat alteration on movement ecology remains understudied.

Studying movement is important because small mammals, particularly habitat specialists and endemics, are most likely to be impacted by disturbances that alter vegetation structure and connectivity. Their smaller body mass and limited perceptual range result in more tortuous paths (Prevedello et al. 2010), influencing various behaviors such as foraging (Vernes and Haydon 2001), homing (Emlen 1969), and exploring their environment (Fordyce et al. 2016). High-severity fire can remove habitat, reduce landscape connectivity, and disincentivize animals from moving through the burned matrix because large gaps increase perceived predation risk (Bakker and Van Vuren 2004; Wilkinson et al. 2013). In addition, low vegetative cover following more severe burns may increase an individual’s perceived risk as exemplified by lower net squared displacement (Hulton VanTassel and Anderson 2018) and foraging activity (Loggins et al. 2019) in burned areas. Actual predation risk also increases with open ground and less vegetation structure (Kotler et al. 1991; Longland and Price 1991; Hovick et al. 2017). Within a “landscape of fear” framework (Laundré et al. 2001), disturbed areas represent heterogeneous areas of high risk within the home range of an individual, and animals may make foraging, movement, and vigilance trade-offs as a function of perceived risk of predation and other threats to life (Gaynor et al. 2019). The resulting altered movement patterns and foraging behaviors have been documented in many species, including rodents (Van Der Merwe and Brown 2008). This trade-off can be quantified behaviorally and used as an assay for potential perceived predation risk in various contexts. When perceived predation risk decreases, rodents have been shown to run in more tortuous paths and decrease their time around shrubs (Lagos et al. 1995). Moreover, movement behavior has implications for foraging search time, ability to respond to predators, and ease of travel (Lawrence 1966; Vernes and Haydon 2001; Bakker 2006). Analysis of fine-scale movement can also reveal patterns of microhabitat selection (McCay 2000), barriers to connectivity (Kuykendall and Keller 2011; Brehme et al. 2013), and space use that have implications for population-level processes—something that abundance alone cannot demonstrate (O’Donnell et al. 2016).

One way to measure animal movement is via path tortuosity, defined as less directional and more convoluted trails. Tortuosity is a useful metric in quantifying how species might respond when fleeing from a predator (Hodges et al. 2014), moving over unfamiliar ground (Bakker 2006), and for indicating patch heterogeneity and vegetation selectivity (Lagos et al. 1995; Bakker 2006; Prevedello et al. 2010). For example, more tortuous movement paths could result from an animal moving within a mosaic of heterogeneous burn severities (Fordyce et al. 2016), and reflect perceived predation risk (Lagos et al. 1995), space use (Hulton VanTassel and Anderson 2018), and food availability via foraging behavior (Vernes and Haydon 2001).

The Mexican Woodrat (*Neotoma mexicana*; hereafter woodrat) is a small mammal found in montane woodlands of Mexico and the southwestern United States (Cornely and Baker 1986). These nocturnal rodents can weigh up to 255 g, are solitary and aggressive, and commonly build their nests in rock crevices (Armstrong et al. 2011). Although an abundance of literature exists on other woodrat species, very little research has focused on the Mexican Woodrat, and most references are in the fields of microbiology and social behavior (Howe 1977, 1978; Maupin et al. 1994; Cajimat et al. 2008). Converse et al. (2006) examined the response of species to fire by measuring changes in density and important habitat components; however, movement ecology and perception of risk following disturbance events are unknown. Thus, the objective of this study was to assess whether burn severity from recent fire influences woodrat movement ecology as they navigate landscapes altered by fire. Previous work indicates that path tortuosity (Lagos et al. 1995; Hodges et al. 2014; Fordyce et al. 2016) and step length (Hodges et al. 2014) can reflect perceived risk in unfamiliar or altered terrain, as would be expected in a landscape of fear (Van Der Merwe and Brown 2008). However, these measures of movement have rarely been studied in relation to altered habitat structure from a disturbance. Here we tested the influence of burn severity, vegetation composition, and structure on movement behavior by translocating woodrats from their territory center across matrices of varying burn severity. We used fluorescent powder tracking (McCay 2000; Kuykendall and Keller 2011; Brehme et al. 2013) to quantify path tortuosity of woodrats over potentially unfamiliar ground in burned versus unburned areas. We also examined microhabitat selection as a function of burn severity.

## Materials and methods

### Ethics statement

Rodents were handled with approval from the University of Arizona Institutional Animal Care and Use Committee and the Arizona Game and Fish Department, and in accordance with the guidelines of the American Society of Mammalogists (Sikes et al. 2016).

### Study site

**Fig. 1.**
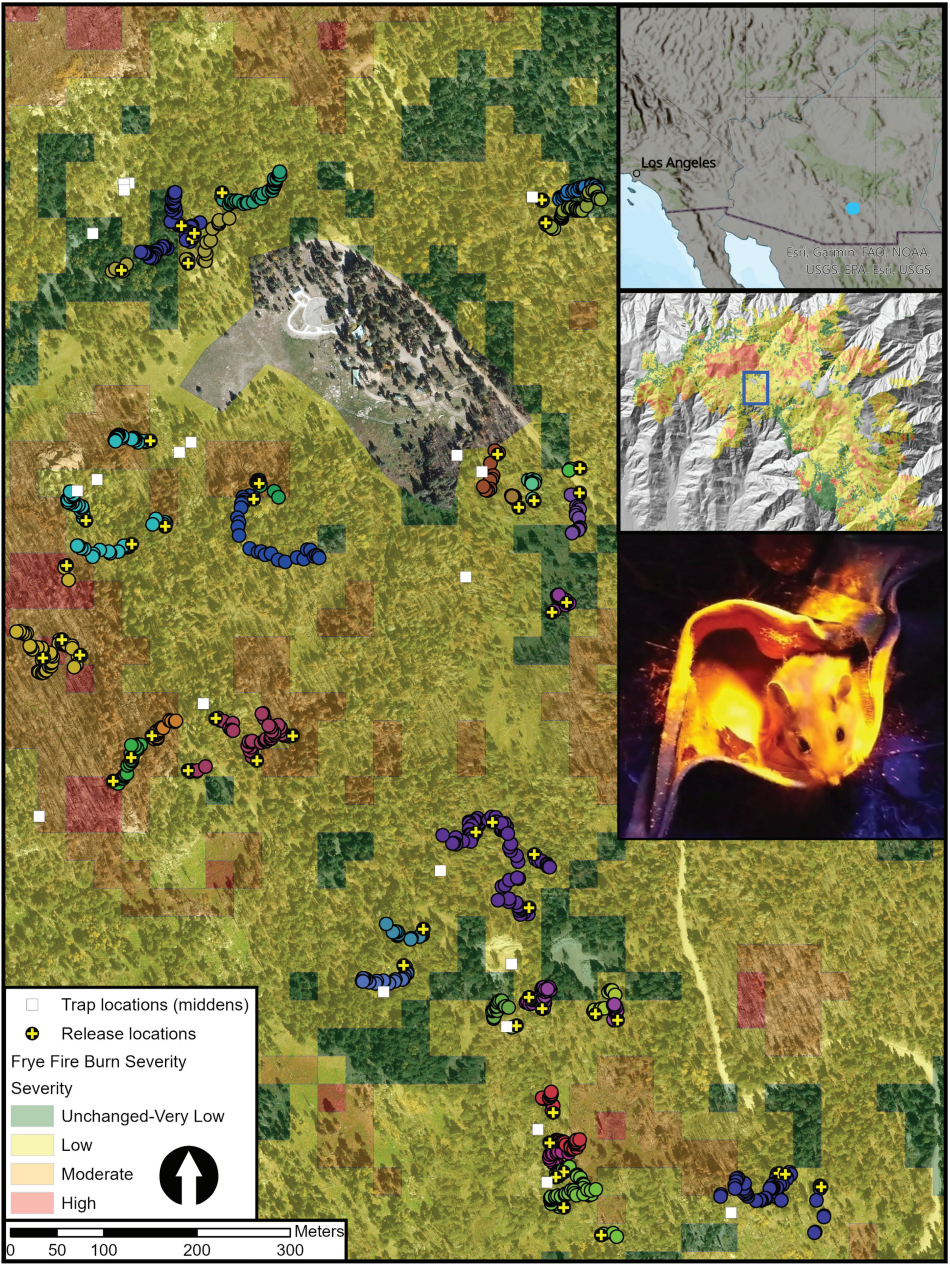
Study area overview in the Pinaleño Mountains of southeastern Arizona, Graham County, Coronado National Forest, United States. Left panel shows study area in detail, where individual Mexican woodrats (*Neotoma mexicana*) were released (crosses) and their subsequent movement paths (filled circles; identified via fluorescent powder tracking) within a matrix of burn severity (semitransparent colored overlay).

The Pinaleño Mountains (Mt. Graham) are the highest within the southern Arizona sky island archipelago, a group of isolated mountain peaks that vary drastically in species composition and climatic conditions with increasing elevation. Overstory vegetation at an elevation of 2,300 to 3,000 m is primarily dominated by Douglas-fir (*Pseudotsuga menziesii*), White Fir (*Abies concolor*), Trembling Aspen (*Populus tremuloides*), Engelmann Spruce (*Picea engelmannii*), and Ponderosa Pine (*Pinus ponderosa*; O’Connor et al. 2017). Mean temperatures range from below freezing in the winter months of December to March, to over 20 °C in the summer months of May to October, and rainfall is heaviest in the summer wet season in July and August (https://raws.dri.edu/cgi-bin/rawMAIN.pl?azACOL). The study area was 1.5 km^2^ and situated within the 19,425 ha burned by the 2017 Frye Fire (Merrick et al. 2021; Fig. 1). Trapping occurred at elevations ranging from 2,880 to 2,925 m.

### Woodrat capture and release

Between June and November 2019, we trapped, translocated, and marked woodrat movement paths in patches of varying burn severity. We identified woodrat middens (territory centers) in rock outcrops based on a hoard of branches, vegetation, and other debris, a bed of fecal pellets (latrine), and a distinct smell. We marked the location in Avenza Maps 3.7.2 (Avenza Systems Inc.) with a georeferenced PDF as our base field map.

To capture woodrats, we used small, 7.62 × 8.89 × 22.86 cm aluminum folding Sherman traps (H.B. Sherman Traps, Tallahassee, Florida) baited with peanut butter and oats and set between 18:00 and 19:00 near the midden center (*n* = 5 traps per site). On average, we trapped at 6 sites per night, totaling 30 traps set per night and 120 traps set per week. We left traps open for an average of 11 h per night, a total of 12,540 trap hours across 38 nights of trapping. Traps were checked between 04:30 and 07:00 h and any captured woodrats were individually marked, dusted in fluorescent powder, and moved a random direction from their midden.

Upon capture, we used Avenza maps to select a random bearing away from the midden of an individual and then moved the animal 50 m in that direction. Although there is no published literature on the home range size of *N. mexicana* to guide our estimates of what may be familiar to individuals of this species, comparisons can be drawn with other woodrat species. White-throated woodrats (*N. albigula*), a species also distributed across Arizona albeit at lower elevations, maintain home ranges of 161 to 486 m^2^ (Macêdo and Mares 1988). If these were perfect circles, they would have a diameter of 14 to 25 m, which would support our assumption of a translocation of 50 m to represent less familiar ground. Woodrats caught multiple times throughout the study were translocated in a different direction on each occasion, and a different bearing was chosen only if the original random bearing traversed a road or dense field of sedge (*Cyperaceae*), grass (*Poaceae*), or fern (*Dennstaedtiaceae*) species, which made powder trails difficult to track. At the selected release location, we placed woodrats in a cloth handling cone (Koprowski 2002), and sexed and individually marked them with a unique numbered tag (National Band & Tag Company) affixed to the ear pinnae. Thereafter, we moved individual woodrats to a 2-gallon plastic storage bag, weighed them with a 300-g Pesola spring scale, and covered them in either pink, orange, or yellow fluorescent powder (TechnoGlow; www.technoglowproducts.com/ultra-glow) by inserting a shaker containing the powder into a small opening at the top of the bag (Lemen and Freeman 1985). The bag was then gently shaken and rotated to saturate the fur in powder. Following powder application, we oriented the woodrat in the direction of its midden and released it. Lastly, we noted the age of an animal (adult or juvenile) based on a 120-g cutoff point obtained from histograms of body mass, and recorded burn severity of the patch over which the woodrat was translocated. Burn severities were classified as severe, moderate, low, or unchanged and obtained from the Burned Area Emergency Response Imagery for the 2017 Frye Fire (https://fsapps.nwcg.gov/baer/home). We pooled the “unchanged” and “low” severity classes into 1 class (low) and the “severe” and “moderate” severity classes into 1 class (high) due to small sample sizes of woodrat territories in those classes within our study area. If another woodrat was caught in the vicinity, or the same woodrat was caught again several days later, we used a different powder color to avoid confusing trails. On the evening following woodrat release, we examined the midden with an ultraviolet flashlight to see if there was evidence that the rodent had returned (i.e. successfully homed).

### Powder trail analysis

We marked powder trails on the evening following woodrat release using an ultraviolet flashlight. We placed a pin flag at each major turning point (McCay 2000), here defined as a directional change equivalent to or greater than 21°—including at the location of release and final place where the powder was last seen. A bearing was taken at each point using a compass, and each step of the trail 1 m or greater was measured to the nearest hundredth of a meter with a tape measure. We recorded understory vegetation based on a line-intercept method along the meter tape and classified vegetation into 1 of 5 categories: logs (fallen trees), branches (woody debris), rocks, bare ground, and dense vegetation (grasses and ferns). Logs were included even if woodrats only traveled along the edge as opposed to directly on it. Vegetation features were all on the same scale (proportions) and only recorded if they spanned at least 1 m of the given step. We used the Bearing and Distance Tool in ArcMap 10.7.1 to reconstruct woodrat movements based on coordinates of the release point and then calculated fractal dimension and squared displacement of these digitized trails (Fig. 1). Trails with a total straight-line distance less than 10 m from beginning to end were excluded from analysis.

### Randomized vegetation transects

We compared vegetation composition along actual woodrat movement paths to randomly generated 50-m transects. We generated a total of 24 random vegetation transects across the study site and again measured vegetation composition using a line-intercept method as previously described, with 12 transects in low-burned areas and 12 in high-burned areas. Location and direction of the transects were determined based on a randomly generated point and bearing. We averaged vegetation proportions for each of the 5 groundcover categories (see above) for both low- and high-burned areas.

### Statistical analysis

We used the “trajr” package for R (McLean and Skowron Volponi 2018) to calculate the fractal dimension and squared displacement of 47 woodrat movement paths, and also included straightness and step length as response variables. Fractal dimension is a measure ranging between 1 and 2 where 1 is a straight line and 2 indicates Brownian motion (Benhamou 2004). Straightness is an alternative approximation for tortuosity and defined as the straight-line distance between start and end point divided by the total trajectory length, where a value of 1 indicates a straight line (Benhamou 2004). Step length (m) refers to a given segment of a woodrat trail. Squared displacement represents the distance between original location and subsequent points along the path (Bastille-Rousseau et al. 2016). We constructed a priori models with each of these measures as the response variable as a function of different intrinsic (sex, age, body mass) and extrinsic (burn severity, vegetation composition) explanatory variables using a generalized linear mixed model, with animal ID as the random effect (Table 1; Supplementary Data SD1). Due to low sample size, we could only include single- or 2-variable models. For those with 2 variables, we focused primarily on interactions between log or bare ground and other predictors because of the documented influence that these 2 vegetative features have on small mammal movement (McMillan and Kaufman 1995; Bakker 2006; Knowlton and Graham 2010). Collinearity among vegetation features was assessed using a correlation matrix, which indicated no significant correlations. We used delta AICc values ≤ 2 to select the top model(s).

**Table 1.** Models for fractal dimension, straightness, step length, and squared displacement for movements of Mexican woodrats (*Neotoma mexicana*) in a mosaic of burned mixed conifer forest, Pinaleño Mountains of southeastern Arizona, United States. See text for parameter definitions.

Null
Burn
Log
Bare ground
Dense vegetation
Rock
Branch
Age
Sex
Mass
Bare ground * log
Dense vegetation * log
Burn * log
Burn * bare ground
Age * log
Age * bare ground
Age * dense vegetation

The *z*-test for proportions described by Byers et al. (1984) was used to compare averages of observed versus expected proportions for the 5 vegetation classes. We compared vegetation composition of used versus random paths separately for low- and high-burned areas. The expected proportions were derived from averaging the proportional representation of each of the 5 vegetation classes from the randomly generated transects (Supplementary Data SD2). We also used a Bonferroni correction on the significance level of α = 0.05 to account for multiple comparisons of 5 vegetation classes. All analyses were conducted in RStudio 4.2.1, except for the *z*-tests, which were conducted in Microsoft Excel.

## Results

### Sample size

**Fig. 2.**
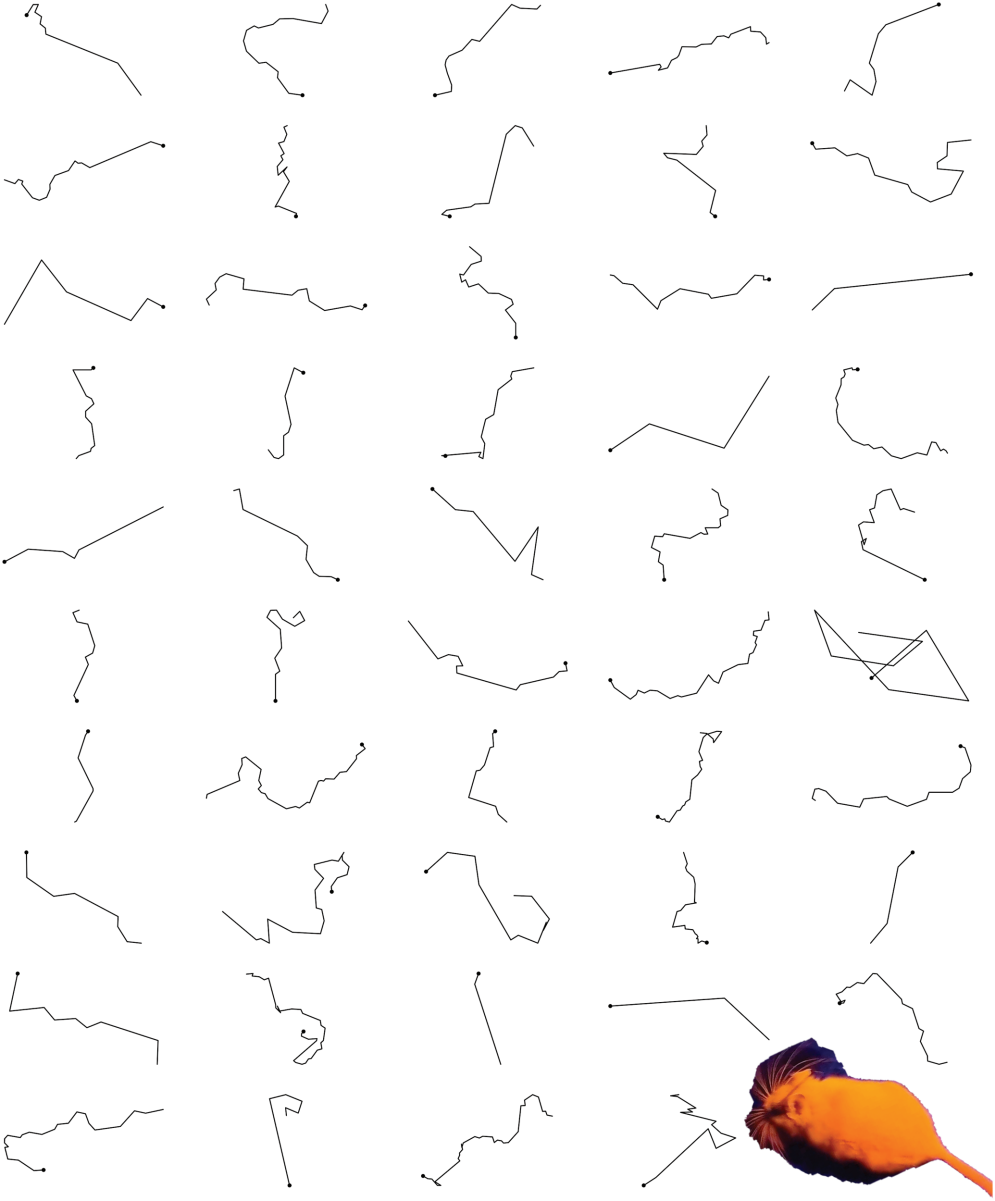
A visual representation of movement trajectories of Mexican woodrats (*Neotoma mexicana*) following translocation across a mosaic of burn severity and ground cover in the Pinaleño Mountains of southeastern Arizona, United States.

We marked a total of 47 trails (Fig. 2) and excluded 1 outlier to achieve normality of residuals in our models. Of the remaining 46 trails, 34 were over low-burned areas and 12 were over high-burned areas. These trails represented movements of 15 females and 13 males. Males and females represented 4 and 8 trails in the high-burned areas, respectively, and 15 and 19 trails in the low-burned areas. All woodrats appeared to successfully return to their nests.

### Woodrat movement

**Fig. 3.**
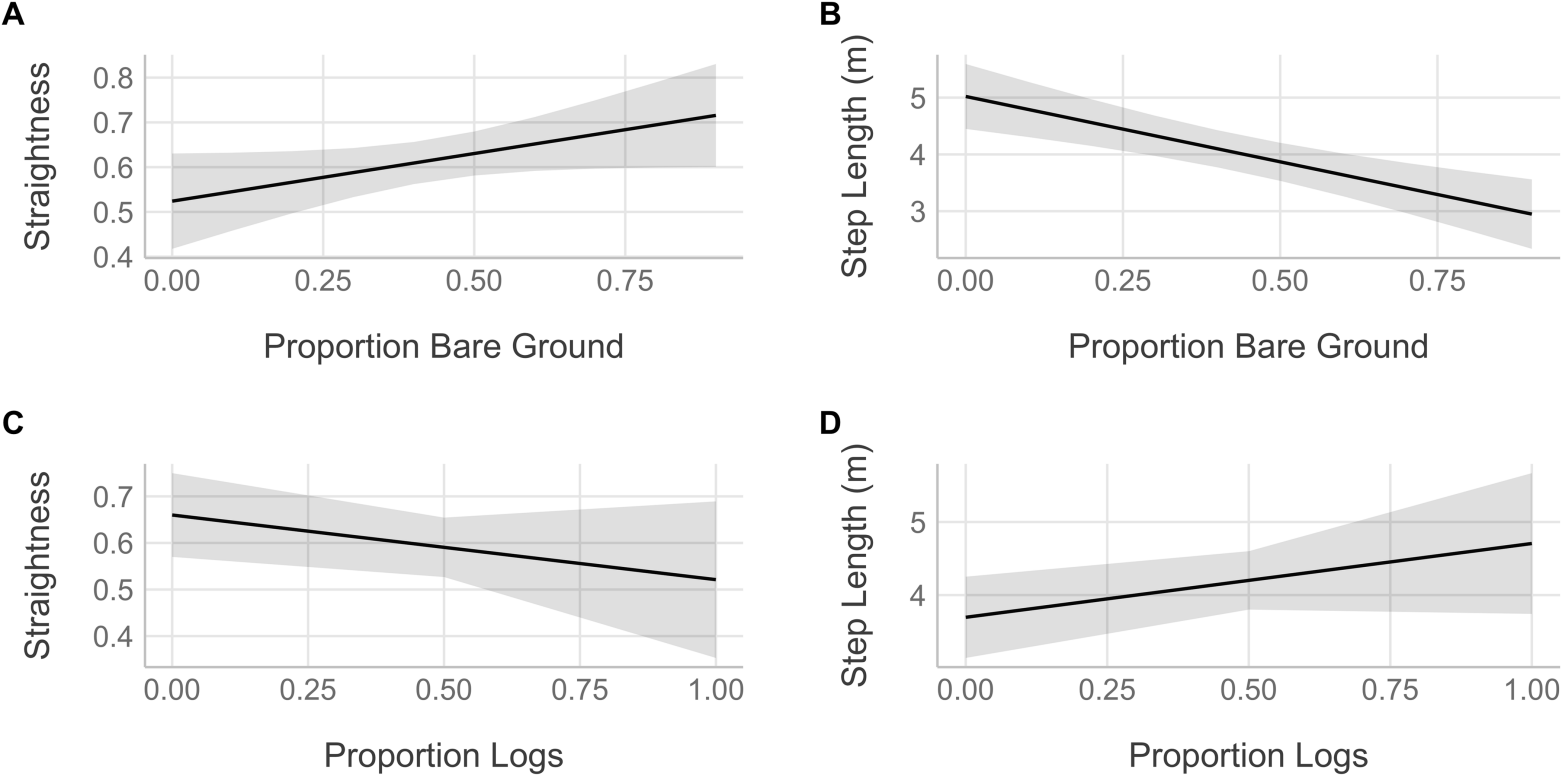
Predicted relationships from univariate models between (A) straightness and bare ground, (B) step length and bare ground, (C) straightness and logs, and (D) step length and logs for movements of Mexican woodrats (*Neotoma mexicana*) in a mosaic of burned mixed conifer forest in the Pinaleño Mountains of southeastern Arizona, United States. A straightness value closer to 1 indicates a straight line.

Among movement indices, bare ground and logs are extrinsic variables that appear to influence woodrat movement behavior. Due to high model selection uncertainty for fractal dimension (Supplementary Data SD3), we refrain from discussing this parameter further and focus the remainder of our discussion on straightness, step length, and squared displacement. Straightness included 3 top competing models that contained bare ground, logs, and burn severity in addition to the null model—although confidence intervals were broad and no effects were significant (Table 2; Fig. 3A and C). The 2 competing models for step length both contained bare ground in an interaction (Table 2). However, given that these interaction terms were insignificant, we also report in Table 2 the effect sizes for bare ground, log, and age separately from noncompeting, univariate models, of which only bare ground was significant (*t* = −4.12, *P* = 0.0002; Table 2; Fig. 3B and D). Lastly, increased proportion of logs resulted in higher squared displacement (*t* = 3.48, *P* = 0.0011) but had no influence on step length (Table 2).

**Table 2.** Competing models (ΔAIC < 2.0) for straightness, step length, and squared displacement for Mexican Woodrat (*Neotoma mexicana*) movements in a mosaic of burned mixed conifer forest, Pinaleño Mountains of southeastern Arizona, United States. We also report results for 3 additional noncompeting, univariate models (see text for explanation). A bolded * indicates that confidence intervals do not overlap 0.

Parameter	Model	Conditional *R*^2^	Variable	Estimate	Standard error
Straightness^a^	Bare ground * log	0.08	Bare ground	0.20	0.16
			Log	−0.05	0.22
			Bare ground * log	−0.08	0.63
	Burn severity^b^	0.06	Burn severity	0.10	0.05
	Null	0	-	-	-
Step length	Bare ground * log	0.69	Bare ground	−1.79*	0.75
			Log	1.04	1.02
			Bare ground * log	−2.98	3.20
	Bare ground * age^c^	0.63	Bare ground	−2.95*	0.66
			Age	−1.24*	0.57
			Bare ground * age	2.13	1.17
	Bare ground^d^	0.66	Bare ground	−2.30*	0.56
	Log^d^	0.39	Log	1.01	0.69
	Age^c^^,^^d^	0.27	Age	−0.32	0.32
Squared displacement	Log * age^c^	0.29	Log	2,375.80*	682.00
			Age	295.40	381.80
			Log * age	−2,618.50*	977.10

^a^A value closer to 1 indicates a straight line.

^b^The category “low” is the reference level.

^c^The category “adult” is the reference level.

^d^Noncompeting model.

### Vegetation

The *z*-test for proportions revealed selection for logs in low-burned patches, albeit by a slim margin, and avoidance of densely growing species in highly burned patches (Table 3).

**Table 3.** Average observed and expected proportions of ground cover classes in low- and high-burned areas in a mosaic of burned mixed conifer forest, Pinaleño Mountains of southeastern Arizona, United States.

		Low		High	
		Observed	Expected	Observed	Expected
Vegetation class	Log	**0.32** ^a^	0.10	0.33	0.17
	Bare ground	0.38	0.58	0.56	0.51
	Branch	0.06	0.08	0.04	0.07
	Rock	0.12	0.02	0.04	0.04
	Dense vegetation	0.12	0.21	**0.01** ^a^	0.10

^a^ **Significant** after Bonferroni correction (α = 0.05/5 = 0.01).

## Discussion

Movement behavior indices such as path tortuosity, step length, and squared displacement reflect how individuals interact with and perceive components of their environment, including perceived predation risk or safety (Lagos et al. 1995). Advances in VHF and satellite technology are providing abundant data on fine spatial and temporal scale space use, behavioral states, and perceived risk for larger species (Nams and Bourgeois 2004; Frair et al. 2005; Coulombe et al. 2006; Valeix et al. 2010; Webb et al. 2011), although a wealth of information can also be provided through cheaper techniques such as fluorescent powder tracking. We examined straightness, step length, and squared displacement in woodrats translocated to patches of high and low burn severity.

Step length and squared displacement varied as a function of vegetation composition and structure. Straightness did not vary significantly with any extrinsic variables (logs, bare ground, burn severity), which was unexpected because differences in structural complexity between low- and high-burned areas would suggest more directional movement in patches with less cover (Stapp and Van Horne 1997; Schooley and Wiens 2003; Knowlton and Graham 2010). Nevertheless, this finding may be attributed to homing behavior—differences in tortuosity between patches might be evident in the daily routine of woodrats (i.e. when foraging) in familiar areas instead of when traversing unfamiliar ground (Lagos et al. 1995; Zollner and Lima 1999; Moriarty et al. 2016). By contrast, step length became shorter with increased bare ground. This outcome is initially counterintuitive because perceived predation risk in small mammals tends to increase with less vegetative cover (Loggins et al. 2019), causing more directional movement (Lagos et al. 1995; Knowlton and Graham 2010) and longer step lengths. The shorter step lengths and less directional movement we observed in areas with high bare ground are likely due to the affinity of a woodrat for using logs for travel through burned areas. The abundance of fallen trees (logs) from historic fire provides plenty of multidirectional runways, and we observed that woodrat trails typically gravitated toward the nearest log instead of traversing an open area. Areas with higher proportions of bare ground generally had lower proportions of logs. Therefore, shorter step length is likely a function of noncontiguous log runways as woodrats traveled the minimum distance required over patches of bare ground to reach the nearest fallen tree. This explanation is reinforced by woodrat selection for logs in low-burned areas, leading as well to the observed higher squared displacement with increased proportions of logs.

Previous studies echo the importance of logs and woody debris for small mammal movement. Bakker (2006), McCay (2000), and McMillan and Kaufman (1995) note that logs provide efficient travel for small mammals. Innes et al. (2007) report that woodrats avoid building nests on ground with mat-forming shrubs, and that logs are a significant predictor of locations of woodrat middens. Logs additionally provide auditory concealment, as running over woody debris or through dense and dry vegetation causes a rustling noise (Barnum et al. 1992; Roche et al. 1999; McCay 2000). Auditory concealment may be particularly important in a heavily burned landscape with little overstory canopy to shield woodrats from avian predators. Moreover, we did not find evidence for selection of logs in heavily burned areas, which could be associated with high numbers of fallen trees in those patches, making it difficult to detect selection. That there was no apparent influence of age or sex on path tortuosity further reinforces that structural complexity and perceived risk may be most important in determining movement parameters.

In addition to selection for logs in low-burned areas, we found that woodrats avoided dense vegetation in high-burned areas. Woodrats may avoid grasses and sedges due to lower visual perception, especially because of the large size of this species and preference to move freely over the landscape. Highly burned areas had the least amount of dense vegetation, yet woodrats may be more vulnerable to predation at those sites (Lawrence 1966; Longland and Price 1991)—thus avoidance of dense vegetation when it is encountered in highly burned sites is heightened. Grasses, sedges, and ferns could also present an obstacle to woodrats as they move over the landscape because of hindrance to mobility, suggesting that movement patterns are primarily influenced by vegetation composition and structure rather than burn severity per se. Woodrats frequently ran around dense patches dominated by *Poaceae* or *Cyperaceae* as opposed to moving through them. Similar results were reported by Beckmann et al. (2002), who found that woodrat middens were unassociated with grasslands though grasses were the dominant vegetation type. Tracked individuals did not avoid dense vegetation in low-burned areas, likely because there is less need for auditory concealment in areas with increased cover.

Whereas most previous research has focused on movement paths during foraging or flight from predators, this work assessed how a forest-dwelling small mammal inhabiting a structurally complex landscape moves over unfamiliar terrain during experimental postfire translocations. Site familiarity confers many advantages including the ability to optimize resource use, access to mates, and heightened ability to avoid predation via escape routes and refugia committed to motor and physical memory (Stamps 1995; Merkle et al. 2014; Oliveira-Santos et al. 2016; Gehr et al. 2020). However, animals are likely to face movement through unfamiliar areas at some point in their lives as is the case during natal dispersal, or because of local depletion of resources, reintroductions and translocations (Ranc et al. 2022), and disturbance events such as fire (Merrick et al. 2021). When faced with novel environments, strategies for navigating efficiently and quickly in 3-dimensional space are critical (Stamps 1995; Oliveira-Santos et al. 2016).

Despite our findings, our study has several limitations, specifically regarding site familiarity. Even if the site of release was beyond the home range of an individual, they still likely had some familiarity with the area from exploratory behavior and known landmarks (Emlen 1969), albeit with insufficient experience for motor learning of escape routes (Van Vuren et al. 1997). That all individuals successfully navigated back to their middens from the release site each night suggests that our translocation distances were within an area familiar to each animal (Van Vuren et al. 1997). Woodrats frequently ran in a wide circle around the point of release prior to orienting in a chosen direction. This was particularly true in heavily burned areas, suggesting individuals spent time considering their surroundings and making informed movement choices rather than exhibiting flight behavior (Bakker 2006). These initial wanderings were also incorporated into analysis, potentially inflating tortuosity values. Moreover, our sample size is low, particularly in highly burned areas. There was an absence of middens in these highly burned regions because both woodrats and their nests perish in fire (Tevis 1956; Simons 1991). However, other small mammal studies with relatively small sample sizes have filled large data gaps about how small mammals make movement decisions and respond to landscape and microhabitat features (e.g. McCay 2000; Bakker 2006; Kuykendall and Keller 2011; Brehme et al. 2013). Most woodrats that were translocated over moderately burned patches in this study originated from middens near the border of low- and moderate-burned areas, suggesting that those individuals may have been displaced from the adjacent lowly burned, and more structurally complex, patches (Simons 1991). Lastly, there are other variables that may influence small mammal movement and microhabitat use, such as cloud cover and moon phase (O’Farrell 1974; Price et al. 1984; Simonetti 1989; Kotler et al. 1991; Wilkinson et al. 2013). In Allegheny woodrats, activity occurred across all moon phases and moonlight illumination explained 7% of variation in woodrat activity, with activity being lowest on full moon nights (Stovall and Hayslette 2013). Across the 22 individual nights represented in our sample, most of our tracking nights occurred during 2 moon phases, gibbous (50%) and crescent (23%), and we did not include moon phase as a random effect in our linear models (see Supplementary Data SD4 for the representation of moon phases in our data).

In an era of unprecedented landscape change globally (Sergio et al. 2018), it is increasingly important to understand how species and individuals within a species respond to altered landscapes and navigate through matrices of nonhabitat, as this response is often species-specific (Kennedy and Marra 2010; Brehme et al. 2013). For many forest-dwelling small mammal species, management actions that preserve fallen trees may be one of the best ways to enhance movement through a heterogeneous matrix of burn severities and facilitate recolonization of quality habitat patches postfire (McMillan and Kaufman 1995; Bakker 2006; Fordyce et al. 2016). For example, Bakker (2006) found that red squirrels (*Tamiasciurus hudsonicus*) translocated across clear-cuts were 8 times more likely to use logs for travel and avoided moving through dense herbaceous and shrub cover. In the same study, 76% of released red squirrels preferentially used the treated half of experimental plots where logs were enhanced on 1 side (Bakker 2006). In addition to underscoring the importance of logs for promoting efficient movement through burned patches, our findings also highlight the importance of efforts to replicate natural fire regimes through prescribed fire or other management strategies to prevent large-scale, high-severity burns that are increasingly common in the western United States (Coop et al. 2020). Ultimately, new insight into how species perceive, learn, and utilize landscape components in the context of disturbance events can help inform management actions that promote population persistence and gene flow.

## Supplementary data

Supplementary data are available at *Journal of Mammalogy* online.

**Supplementary Data SD1.**—Movement response variables in addition to covariate data that were used in statistical modeling.

**Supplementary Data SD2.**—Results from randomized vegetation transects that were used to obtain expected proportions of vegetation cover.

**Supplementary Data SD3.**—Full AICc output of models fit to fractal dimension, straightness, step length, and squared displacement.

**Supplementary Data SD4.**—Bar graph of the different moon phases represented in our sample.

gyad117_suppl_Supplementary_Datas_SD1

gyad117_suppl_Supplementary_Datas_SD2

gyad117_suppl_Supplementary_Datas_SD3

gyad117_suppl_Supplementary_Datas_SD4

## Data Availability

All data generated or analyzed during this study are included in the additional files.
