# Supplementary material for: Movement response of small mammals to burn severity reveals importance of microhabitat features
Source: J Mammal. 2023 Dec 24;105(1):157–67. doi: 10.1093/jmammal/gyad117 (PMC11647521; doi:10.1093/jmammal/gyad117)
Supplement: gyad117_suppl_Supplementary_Datas_SD3 [file gyad117_suppl_supplementary_datas_sd3.docx]

AICc table for fractal dimension

| Modnames | K | Conditional R^2^ | AICc | Delta_AICc | ModelLik | AICcWt | LL | Cum.Wt |
| --- | --- | --- | --- | --- | --- | --- | --- | --- |
| null | 2 | 0.9567 | -168 | 0.00 | 1.000 | 0.1687 | 87 | 0.17 |
| sex | 3 | 0.9568 | -167 | 0.58 | 0.748 | 0.1262 | 88 | 0.29 |
| burn * logs | 5 | 0.9694 | -167 | 0.97 | 0.617 | 0.1041 | 91 | 0.40 |
| dense veg | 3 | 0.9615 | -166 | 1.35 | 0.509 | 0.0858 | 88 | 0.48 |
| burn severity | 3 | 0.9572 | -166 | 1.75 | 0.416 | 0.0702 | 88 | 0.56 |
| branch | 3 | 0.9593 | -166 | 1.78 | 0.410 | 0.0691 | 88 | 0.62 |
| age cutoffs | 3 | 0.9544 | -166 | 1.93 | 0.381 | 0.0643 | 87 | 0.69 |
| dense.veg * log | 5 | 0.9558 | -166 | 1.99 | 0.369 | 0.0623 | 90 | 0.75 |
| bg | 3 | 0.9584 | -166 | 2.04 | 0.360 | 0.0608 | 87 | 0.81 |
| rock | 3 | 0.9567 | -165 | 2.38 | 0.304 | 0.0513 | 87 | 0.86 |
| logs | 3 | 0.9569 | -165 | 2.38 | 0.304 | 0.0512 | 87 | 0.91 |
| mass | 3 | 0.9576 | -165 | 2.39 | 0.302 | 0.0510 | 87 | 0.97 |
| burn * bg | 5 | 0.9610 | -162 | 5.47 | 0.065 | 0.0109 | 88 | 0.98 |
| age.cutoffs * dense.veg | 5 | 0.9576 | -162 | 6.09 | 0.048 | 0.0080 | 88 | 0.98 |
| age.cutoffs * bg | 5 | 0.9563 | -161 | 6.69 | 0.035 | 0.0059 | 88 | 0.99 |
| age.cutoffs * logs | 5 | 0.9551 | -161 | 6.90 | 0.032 | 0.0054 | 88 | 1.00 |
| bg * logs | 5 | 0.9588 | -161 | 7.16 | 0.028 | 0.0047 | 87 | 1.00 |

AICc table for straightness

| Modnames | K | Conditional R^2^ | AICc | Delta_AICc | ModelLik | AICcWt | LL | Cum.Wt |
| --- | --- | --- | --- | --- | --- | --- | --- | --- |
| bg * logs | 5 | 0.08038 | -29 | 0.00 | 1.000 | 0.1905 | 19 | 0.19 |
| burn severity | 3 | 0.06405 | -28 | 0.39 | 0.821 | 0.1565 | 19 | 0.35 |
| null | 2 | 0 | -28 | 0.97 | 0.616 | 0.1173 | 17 | 0.46 |
| burn * bg | 5 | 0.1348 | -27 | 2.03 | 0.363 | 0.0691 | 20 | 0.53 |
| logs | 3 | 0.02836 | -26 | 2.08 | 0.354 | 0.0674 | 18 | 0.60 |
| rock | 3 | 0.02329 | -26 | 2.31 | 0.315 | 0.0599 | 18 | 0.66 |
| burn * logs | 5 | 0.1291 | -26 | 2.32 | 0.313 | 0.0597 | 20 | 0.72 |
| sex | 3 | 0.01426 | -26 | 2.73 | 0.256 | 0.0487 | 17 | 0.77 |
| branch | 3 | 0.005698 | -25 | 3.12 | 0.210 | 0.0401 | 17 | 0.81 |
| dense veg | 3 | 0.005119 | -25 | 3.14 | 0.208 | 0.0396 | 17 | 0.85 |
| age cutoffs | 3 | 0.003047 | -25 | 3.24 | 0.198 | 0.0378 | 17 | 0.89 |
| mass | 3 | 0.0002805 | -25 | 3.36 | 0.186 | 0.0355 | 17 | 0.92 |
| age.cutoffs * bg | 5 | 0.09217 | -24 | 4.20 | 0.123 | 0.0234 | 19 | 0.95 |
| dense.veg * log | 5 | 0.08162 | -24 | 4.72 | 0.095 | 0.0180 | 19 | 0.96 |
| bg | 3 | 0.07218 | -24 | 4.78 | 0.092 | 0.0175 | 19 | 0.98 |
| age.cutoffs * dense.veg | 5 | 0.06985 | -23 | 5.29 | 0.071 | 0.0135 | 19 | 0.99 |
| age.cutoffs * logs | 5 | 0.03330 | -22 | 7.03 | 0.030 | 0.0057 | 18 | 1.00 |

AICc table for step length

| Modnames | K | Conditional R^2^ | AICc | Delta_AICc | ModelLik | AICcWt | LL | Cum.Wt |
| --- | --- | --- | --- | --- | --- | --- | --- | --- |
| bg * logs | 5 | 0.6863 | 125 | 0.00 | 1.00000 | 0.52466 | -58 | 0.52 |
| age.cutoffs * bg | 5 | 0.6319 | 125 | 0.87 | 0.64565 | 0.33875 | -56 | 0.86 |
| bg | 3 | 0.6647 | 129 | 4.13 | 0.12676 | 0.06651 | -57 | 0.93 |
| burn * bg | 5 | 0.6605 | 130 | 5.11 | 0.07766 | 0.04074 | -58 | 0.97 |
| dense.veg * log | 5 | 0.6035 | 133 | 8.89 | 0.01175 | 0.00616 | -60 | 0.98 |
| sex | 3 | 0.3225 | 134 | 9.76 | 0.00758 | 0.00398 | -63 | 0.98 |
| null | 2 | 0.3015 | 134 | 9.89 | 0.00711 | 0.00373 | -64 | 0.98 |
| rock | 3 | 0.3172 | 135 | 10.11 | 0.00637 | 0.00334 | -63 | 0.99 |
| logs | 3 | 0.3862 | 135 | 10.28 | 0.00585 | 0.00307 | -63 | 0.99 |
| branch | 3 | 0.2665 | 136 | 11.14 | 0.00381 | 0.00200 | -63 | 0.99 |
| age cutoffs | 3 | 0.2697 | 136 | 11.35 | 0.00342 | 0.00180 | -63 | 0.99 |
| burn severity | 3 | 0.3061 | 136 | 11.74 | 0.00282 | 0.00148 | -64 | 1.00 |
| mass | 3 | 0.3099 | 137 | 12.10 | 0.00236 | 0.00124 | -64 | 1.00 |
| dense veg | 3 | 0.2891 | 137 | 12.29 | 0.00215 | 0.00113 | -64 | 1.00 |
| age.cutoffs * logs | 5 | 0.3934 | 138 | 13.52 | 0.00116 | 0.00061 | -62 | 1.00 |
| age.cutoffs * dense.veg | 5 | 0.1073 | 139 | 14.05 | 0.00089 | 0.00047 | -62 | 1.00 |
| burn * logs | 5 | 0.3844 | 139 | 14.73 | 0.00063 | 0.00033 | -63 | 1.00 |

AICc table for squared displacement

| Modnames | K | Conditional R^2^ | AICc | Delta_AICc | ModelLik | AICcWt | LL | Cum.Wt |
| --- | --- | --- | --- | --- | --- | --- | --- | --- |
| age.cutoffs * logs | 5 | 0.2905 | 741 | 0.0 | 1.00000 | 0.83193 | -364 | 0.83 |
| age cutoffs | 3 | 0.09764 | 747 | 5.7 | 0.05821 | 0.04843 | -369 | 0.88 |
| logs | 3 | 0.05228 | 749 | 7.9 | 0.01928 | 0.01604 | -370 | 0.90 |
| null | 2 | 0 | 749 | 7.9 | 0.01915 | 0.01593 | -371 | 0.91 |
| mass | 3 | 0.04851 | 749 | 8.1 | 0.01762 | 0.01466 | -370 | 0.93 |
| age.cutoffs * dense.veg | 5 | 0.1462 | 750 | 8.4 | 0.01523 | 0.01267 | -368 | 0.94 |
| dense veg | 3 | 0.04170 | 750 | 8.4 | 0.01501 | 0.01249 | -370 | 0.95 |
| rock | 3 | 0.03334 | 750 | 8.8 | 0.01234 | 0.01027 | -371 | 0.96 |
| branch | 3 | 0.02216 | 751 | 9.3 | 0.00953 | 0.00793 | -371 | 0.97 |
| age.cutoffs * bg | 5 | 0.1228 | 751 | 9.6 | 0.00827 | 0.00688 | -368 | 0.98 |
| sex | 3 | 0.01412 | 751 | 9.7 | 0.00793 | 0.00659 | -371 | 0.98 |
| burn severity | 3 | 0.003504 | 751 | 10.2 | 0.00623 | 0.00518 | -371 | 0.99 |
| bg | 3 | 6.5657e-05 | 752 | 10.3 | 0.00576 | 0.00479 | -371 | 0.99 |
| dense.veg * log | 5 | 0.08403 | 753 | 11.5 | 0.00312 | 0.00259 | -369 | 1.00 |
| burn * logs | 5 | 0.06794 | 754 | 12.3 | 0.00211 | 0.00175 | -370 | 1.00 |
| bg * logs | 5 | 0.06034 | 754 | 12.7 | 0.00175 | 0.00146 | -370 | 1.00 |
| burn * bg | 5 | 0.005584 | 757 | 15.2 | 0.00049 | 0.00041 | -371 | 1.00 |
