# Supplementary figures and images for: Movement response of small mammals to burn severity reveals importance of microhabitat features
Source: J Mammal. 2023 Dec 24;105(1):157–67. doi: 10.1093/jmammal/gyad117 (PMC11647521; doi:10.1093/jmammal/gyad117)

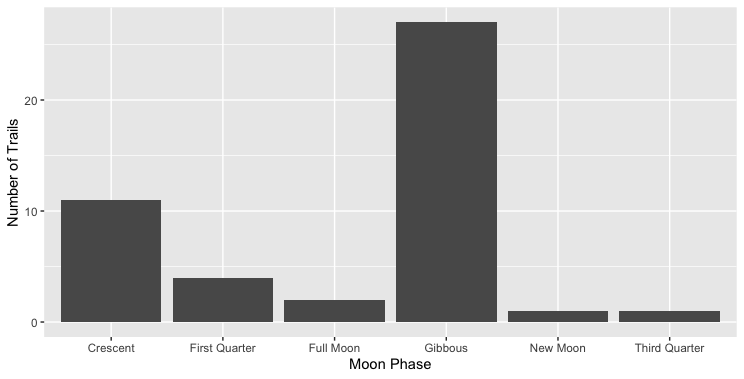

Supplement: gyad117_suppl_Supplementary_Datas_SD4 [file gyad117_suppl_supplementary_datas_sd4.docx]
